# Sustaining attention in affective contexts during adolescence: age-related differences and association with elevated symptoms of depression and anxiety

**DOI:** 10.1080/02699931.2024.2348730

**Published:** 2024-05-07

**Authors:** D. L. Dunning, J. Parker, K. Griffiths, M. Bennett, A. Archer-Boyd, A. Bevan, S. Ahmed, C. Griffin, L. Foulkes, J. Leung, A. Sakhardande, T. Manly, W. Kuyken, J.M.G. Williams, S.-J. Blakemore, T. Dalgleish

**Affiliations:** a Medical Research Council Cognition and Brain Sciences Unit, Cambridge University, Cambridge, UK; b Health Research Methods Unit, University of Hertfordshire, Hatfield, UK; c Institute of Cognitive Neuroscience, University College London, London, UK; d School of Psychology and Language Sciences, University College London, London, UK; e Department of Psychiatry, University of Oxford, Oxford, UK; f Department of Psychology, Cambridge University, Cambridge, UK; g Cambridgeshire and Peterborough NHS Foundation Trust, Cambridge, UK

**Keywords:** Sustained attention, affect, depression, anxiety, adolescence

## Abstract

Sustained attention, a key cognitive skill that improves during childhood and adolescence, tends to be worse in some emotional and behavioural disorders. Sustained attention is typically studied in non-affective task contexts; here, we used a novel task to index performance in affective versus neutral contexts across adolescence (*N *= 465; ages 11–18). We asked whether: (i) performance would be worse in negative versus neutral task contexts; (ii) performance would improve with age; (iii) affective interference would be greater in younger adolescents; (iv) adolescents at risk for depression and higher in anxiety would show overall worse performance; and (v) would show differential performance in negative contexts. Results indicated that participants performed more poorly in negative contexts and showed age-related performance improvements. Those at risk of depression performed more poorly than those at lower risk. However, there was no difference between groups as a result of affective context. For anxiety there was no difference in performance as a function of severity. However, those with higher anxiety showed less variance in their reaction times to negative stimuli than those with lower anxiety. One interpretation is that moderate levels of emotional arousal associated with anxiety make individuals less susceptible to the distracting effects of negative stimuli.

Sustained attention is the ability to maintain goal-directed focus on a stimulus or an activity over time, even in the face of competing task-irrelevant information. Individual differences in sustained attention have been linked to performance variability in a host of other processes including learning and memory (Cowan, 1998).

Sustained attention is often measured using vigilance tasks that involve a participant monitoring a stream of stimuli over an extended period for the occurrence of rare targets, to which they should respond. Poorly maintained attention can be indexed by missed targets or by slowing or variation in reaction time (RT) to those targets (e.g. Stuss et al., 1989). Robertson et al. (1997) argued that increasing automaticity of the response to the rare targets may offset task sensitivity to declining or lapsing attention. Accordingly, in the Sustained Attention to Response Task (SART), Robertson et al. reversed the standard vigilance contingencies by asking participants to respond to frequent, regularly presented non-targets (“Go” trials) while *withholding* responses to rare targets (“No-go” trials). In this manner, they argued, it would be the frequent non-target response that would tend to become automatic, leading to errors on the target trials unless the participants maintained active attentional control over their responses. An advantage of the SART, compared with vigilance measures in which responses are only made to rare targets, is the additional information from reaction times to the vast majority of stimuli that are go trials (Robertson et al., 1997). It has been argued that poorly maintained attention to the task is associated with greater variability in these go RTs, sometimes reflecting slow and considered responses to each stimulus and at other times appearing to be triggered by the *anticipated* occurrence of the next stimulus within the regularly timed sequence.

The SART was the model for our *Affective* Sustained Attention to Response Test (aSART). As with the SART, participants were shown a regularly paced, random sequence of single digits with the instruction to press the same key after each digit except after the nominated no-go target (the number “3”) that occurred with a low probability. To create the affective conditions, participants were exposed to a blended stream of task-irrelevant sounds during performance (International Affective Digitized Sounds (IADS); Bradley & Lang, 2007). In one condition the sounds were negatively valenced (e.g. baby crying) and in the other, neutral (e.g. crowd murmur). The relatively straightforward idea was that, if negative auditory distractions were more effective in intruding into the task, this would then cause a relative increase in errors of commission (pressing for rare targets) and perhaps induce greater variability in RT, relative to the neutral condition. Consequently, how good participants are at ignoring this negative (relative to neutral) interference represents an index of their affective sustained attention.

## Sustained attention and emotional reactivity across adolescence

Performance on typical, non-affective, sustained attention tasks improves rapidly between the ages of around 10 and the middle teenage years, reaching adult levels at around 15 years of age (Lin et al., 1999). This improvement is usually seen in terms of better accuracy and a decrease in RT variability (and indeed RT), in line, one could argue, with a shift towards a slower, more cautious response strategy.

In addition to less well-developed sustained attention skills, younger adolescents also tend to have increased frequency and intensity of emotions and show greater emotional reactivity to negative stimuli than older adolescents (see Bailen et al., 2019, for a review), suggesting that sustaining attention in affective or “hot” contexts may be particularly difficult for adolescents.

The first motivation behind developing the aSART was therefore to provide a laboratory measure of any decrement in the ability to sustain attention in negative affective, relative to neutral, contexts that could be used to investigate age-related changes in sustained attention in both neutral and affective contexts, across the developmentally sensitive period of adolescence. This, we submit, is important for several reasons. First, it can begin to tell us about the interplay between sustained attention and the negatively affective nature of the environment in which the attention is deployed. Second, prior research on the nature of age-related changes in the impact of affective context on cognitive performance, including both attentional and broader executive tasks, suggests that the trajectories vary as a function of cognitive domain. Some facets showing a linear relationship with affective impact – sometimes declining and sometimes increasing with age – others show no age-related effects, and yet others suggest a non-linear pattern (see Schweizer et al., 2020, for a review). Elucidating the nature of the age-related profile for sustained attention in affective contexts is therefore key in helping to build this complex picture. Finally, if the aSART proves sensitive as an index of affective influences on sustained attention it can also then be used as an assay of individual differences as a function of mental health status, providing a window into how this important cognitive skill can become disrupted in those vulnerable to or suffering from emotional disorders such as depression or anxiety.

## Sustained attention and mental health

Problems with concentration are a hallmark feature of depression. It is therefore unsurprising that deficits in sustained attention have been shown extensively in depressed adults. The precise mechanistic relationship between sustained attention and depression is unknown, but one possibility is the excessive rumination associated with depression places a demand on cognitive resources thus limiting the ability to effectively sustain concentration on extrinsic neutral stimuli (van Vugt & van der Velde, 2018). Although the link between depression and sustained attention is well established in adults, it is less well defined in depressed youth populations. There is a suggestion that depressed adolescents make more errors of both commission (responding to non-targets) and omission (failing to respond to targets) than non-depressed adolescents, but evidence for differences in RT and RT variability are equivocal. We know very little about any age-related changes in these relationships in this developmental period as most studies have not systematically compared different age groups.

There are very few studies examining sustained attention in affective contexts in depressed adolescents. One study showed no significant differences between a group of depressed adolescents and a group of healthy controls on a short Go/No-go vigilance task, when priming responses with either happy or sad faces prior to trial onset (Colich et al., 2016). Relatedly, and also using shorter Go/No-go task vigilance designs, there have been mixed results when the targets themselves have been emotionally salient. Kyte et al. (2005) found that depressed adolescents made fewer commission errors when the targets were “sad” compared to “happy” words. Ladouceur et al. (2006) showed that, although there were no differences between a depressed group and low-risk controls in error rate, depressed youth responded significantly faster to sad than happy faces as targets. Taken together, these studies show an inconsistent pattern of results. This could be a consequence of the typically small sample sizes used in the studies as well as differences in task and methodology. Crucially, none of these studies has used the “reverse methodology” of the aSART where targets require a no-go response.

To our knowledge, there are no studies of sustained attention in those characterised as *at risk* of depression in any age group. Elucidating whether there are cognitive markers of this depressive vulnerability that play out in affective contexts therefore seems important, especially given the potential of restorative cognitive training to mitigate risk (see Motter et al., 2016 for a review). Although there are no studies examining sustained attention and depression risk in adolescence, studies have examined other aspects of attention and again results are mixed. On the one hand, it has been shown that youth at risk of depression exhibit attentional *avoidance* of negative socio-affective stimuli (e.g. Harrison & Gibb, 2015). This suggests that sustained attention in affective contexts might be superior in an at-risk sample due to an enhanced ability to ignore negative distractors across time. On the other hand, some studies in adolescents deemed at risk of depression suggest that there is *increased* attention to negative information in these vulnerable samples (e.g. Hankin et al., 2010).

The second motivation of the present study, in the context of these equivocal prior findings, was therefore to utilise the aSART to examine whether sustained attention in affective contexts was better or worse in those adolescents at elevated risk of mental health problems. First, we examine sustained attention performance in those at risk of depression (based on above-cut-off scores on a standard depression measure), relative to their low-risk peers, and to clarify any age-related changes in this relationship. Second, we included a measure of anxiety because of the well-established overlap between trait anxiety and depression in adolescents. There has been very little work on trait anxiety and measures of sustained attention performance in adolescents. Forster and colleagues showed high-trait anxious young adults had slower RTs than those with low-trait anxiety (Forster et al., 2015). However, another study showed no differences between high – and low-trait anxiety on either error rate or RT (Righi et al., 2009).

We consequently administered the aSART, along with robustly validated self-report assessments of depressive symptoms and depression risk, and of anxiety to a large sample of adolescents (*N *= 485) aged 11 to 18 years.

## Hypotheses

For all of the following we chose commission errors and RT variance as the key outcomes. In this respect, better performance on the aSART equates to reduced commission errors and RT variance. The choice to focus on these variables, rather than omission errors and RT, was made as the SART was originally designed with errors of commission as the key variable of interest; this is because the task sets up a response tendency to the very frequent go-targets that must be actively resisted to increase the chance of withholding responses to rare no-go targets (Robertson et al., 1997). While some reports on the SART have focussed on errors of omission these omissions generally occur at such a low rate in the general population that they are difficult to interpret (e.g. potentially occurring as a transient distraction in response to an error of commission). In terms of RT variability versus overall RT, a general finding in the SART literature is that RTs tend to speed up as a participant’s responses become increasingly “driven” by the regular onset of the stimuli rather than attentive processing of each stimulus with respect to what response should be made (participants have pressed before they have time to process) – hence there is a speed-accuracy trade off. Mean RT is therefore generally seen as interpretable – as it could be comprised of very fast “inattentive” responding and slow “attentive” responding or a much more consistent attentionally regulated style. The variability measure is therefore argued to better capture this tendency to drift into task driven responding and then correct into attentive responding vs. consistent style (Manly et al., 1999, 2000; Robertson et al., 1997). Full omission and RT data were included in the supplementary materials for completeness. Our hypotheses were as follows:

1. That performance would be poorer in the negative aSART condition versus the neutral condition, across the sample as a whole.
2. That overall performance on the aSART (irrespective of the valence of the task condition) would be associated with older age across adolescence.
3. That the influence of affective context, i.e. a relative decrement in aSART performance in the negative versus neutral condition, would be greater for younger adolescents relative to their older peers.
4. Adolescents deemed to be at risk for depression according to cut-offs on a measure of depression (hypothesis 4a), or with elevated symptoms of anxiety (hypothesis 4b), would show *overall* worse performance on the aSART relative to those deemed to be lower risk of depression or with lower symptoms of anxiety.
5. Based on the extant literature, we had a non-directional hypothesis that there would be a differential effect of affective context, i.e. a relative difference in aSART performance in affective versus neutral conditions, in those adolescents deemed at risk of depression, compared with their lower risk peers (hypothesis 5a), and in those with higher levels of anxiety (hypothesis 5b), compared to those with lower levels.
6. That any differential effect of affective context in those at risk of depression (hypothesis 6a) and higher in ratings of anxiety (hypothesis 6b) would be greater in younger, relative to older, adolescents.

## Method

Four hundred and eighty-five participants (320 females) aged 11–18 years (*M* = 14.40, SD = 1.80) were recruited from 15 schools and colleges in Greater London and Cambridge (U.K.). Further details about the recruitment process can be found in the Supplemental Materials section, and the demographic, non-verbal IQ and mental health composition of the sample are provided in Table 1 of the Results section.

**Table 1.** Means, standard deviations and range of scores of the main task variables.

	*M* (SD)	Range
aSART		
Commission errors negative condition (raw scores)	16.67 (6.49)	0–30
Commission errors neutral condition (raw scores)	16.24 (6.38)	0–30
RT variance negative condition (ms)	0.33 (.13)	0.11–0.92
RT variance neutral condition (ms)	0.32 (.12)	0.12–0.80
IQ (standard scores)	113.23 (17.05)	76–158
Depression (raw scores)	17.33 (10.49)	0–56
Anxiety (raw scores)	12.96 (7.93)	0–40

Note: aSART: Affective Sustained Attention to Response Task; RT: Reaction time.

### Measures

#### Affective Sustained Attention to Response Test (aSART)

**Figure 1.**
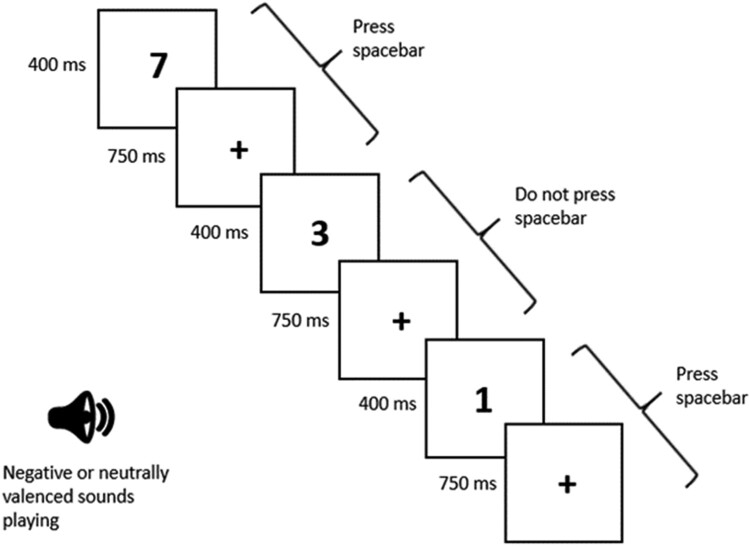
Example of aSART trial sequence.

The aSART was programmed in E-Prime version 2.0 (Schneider et al., 2002) and adapts the original SART through the introduction of different auditory background stimuli – affective versus neutral – to evaluate whether attentional lapses vary as a function of affective context. Apart from the addition of background stimuli, the aSART was identical to the original SART. Both were computer-administered tasks that involved the withholding of key presses to rare (one in nine) targets presented on the screen. Specifically, targets were drawn from the numbers 1–9 and were presented one digit at a time. The participant was simply asked to respond to the appearance of each digit by pressing the space bar (“Go” trials). The exception to this was when the number “3” appeared, to which no response should be made (“No-go” trials). For the aSART, the response window was 1150 milliseconds (ms) – each digit was on screen for 400 ms, followed by a mask (a fixation cross) for 750 ms (see Figure 1). While completing the task, in a within-subjects design, participants listened to a continuous background stream of either neutral- or negative-valence sounds through headphones. The 540 trials were divided into six blocks of 90 trials each. In three of the blocks, participants heard a stream of different negative sounds (e.g. an alarm clock going off, baby crying, etc.) and in the other three blocks they heard a stream of different affectively neutral sounds (e.g. crowd murmur, chickens clucking, etc.). The six blocks were randomly presented. The sounds were taken from the International Affective Digitized Sounds (IADS) corpus (a library of sounds pre-rated for valence and arousal by college attending adults; Bradley & Lang, 2007). Further details of the aSART including a list of all sounds used, along with the adult and adolescent valence ratings from an unreported pilot study (Table S1), can be found in the Supplementary materials.

Five-hundred and forty trials were presented, 60 of which were no-go trials, over a period of 12 mins.

The key outcome variables for the present study were commission errors and RT variance. In addition, we computed indices to measure the effect of affective context by subtracting scores on the key aSART outcome variables in the neutral condition from scores in the negative condition, such that larger scores represented a bigger influence of affective context.

#### Center for Epidemiologic Studies Depression Scale (CES-D)

The CES-D (Radloff, 1977) is a 20-item self-report measure in which participants were asked to rate how often over the past week they had experienced symptoms associated with depression. Responses are summed and scores ranged from 0 to 60, with higher scores indicating greater depressive symptoms. The clinical cut-off for being at risk of depression is a raw score of 16.

#### Revised Children's Anxiety and Depression Scale (RCADS)

The RCADS (Chorpita et al., 2005) is a 15-item self-report questionnaire that measures symptoms of anxiety and low mood The items in the RCADS are summed to give a total score ranging from 0–40 with greater scores indicating greater symptoms of anxiety.

#### Cattell Culture Fair Intelligence Test (CCFIT)

The CCFIT (Cattell, 1963) is a measure of non-verbal intelligence that minimises sociocultural and environmental influences. A paper and pencil version of Scale 2 Form A of the test was used. This comprised four timed subtests that consisted of questions involving the relationships between pictures of abstract geometric shapes. Correct responses were summed, and age-appropriate standard scores were calculated based on a set of existing norms.

Further details on the CES-D, RCADS and CCFIT can be found in Supplemental Materials.

### Procedure

Participants were tested in small groups, supervised by at least two researchers. Before commencing the aSART, participants were asked to put on a set of over-ear headphones, given a set of instructions (see Supplementary Materials for full instructions) and completed a series of practice trials. The aSART took around 12 mins to complete.

## Results

### Participants

Data from 31 participants were excluded from the analysis, either because they did not complete the aSART task (*n* = 8), the CES-D (*n *= 12), the RCADS (*n* = 6) or the CCFIT (*n *= 3), or they scored <70, suggestive of cognitive difficulties, on the CCFIT (*n *= 2). In addition, those with omission errors more than three standard deviations from the mean (*M* = 29.42, *SD* = 25.35) were treated as outliers and excluded from the analysis (*n *= 14). This decision was taken as omitting responses to go trials on a large scale (approximately 100+) artificially inflates performance on no-go trials, meaning fewer commission errors. The exclusions gave an analysis sample of 446 participants (293 females, 153 males); *M* = 14.42 years, *SD* = 1.82 years, age range 11.20–18.50 years.

### aSART performance

Data for the analysis sample on all metrics of aSART for both the affective and neutral conditions are presented in Table 1. Here we report analyses on the two core aSART outcomes of commission errors and RT variance.

#### Hypothesis one

To examine whether aSART performance would be poorer in negative vs. neutral contexts, we analysed within-participant differences between affective conditions using repeated-measures general linear models. In support of our hypothesis, results showed that, overall, participants made significantly more commission errors, *F* (1, 445) = 5.63, *p* < .05, Cohen’s *d *=* *.07*, CI *=* *0.74–0.79, and showed significantly greater RT variance, *F* (1, 445) = 7.50, *p *< .01, *d *=* *.08*, CI *=* *.003–0.02 in the negative versus neutral aSART condition.

### Age-related differences in aSART performance

Zero-order correlations between the independent variables across the analysis sample (Table 2) revealed significant associations between age & gender, age & CES-D, and gender & CES-D. These variables were therefore included as covariates in subsequent analyses.

**Table 2.** Correlations between independent variables.

	Gender	IQ	CES-D	Anxiety
Age (years)	−.18**	.01	.19**	.02
Gender		.08	−.23**	−.22**
IQ (standard score)			.01	.03
Depression (raw score)				.62**

**P < .01.

#### Hypothesis two

In support of our second hypothesis, that performance on the aSART would show an age-related improvement across adolescence, linear regressions (adjusting for gender and CES-D scores), showed that older participants made fewer commission errors ($R_{\mathrm{adjusted}}^{2}$ = .095, *F* (3, 440) = 15.26, *p *< .001, *β *=* −.25* −.22, *p* < .001), and showed lower RT variance ($R_{\mathrm{adjusted}}^{2}$ = .0.10, *F* (3, 440) = 17.86, *p *< .001, *β* = −.32, *p *< .001), than younger participants, with clear linear profiles.

#### Hypothesis three

**Figure 2.**
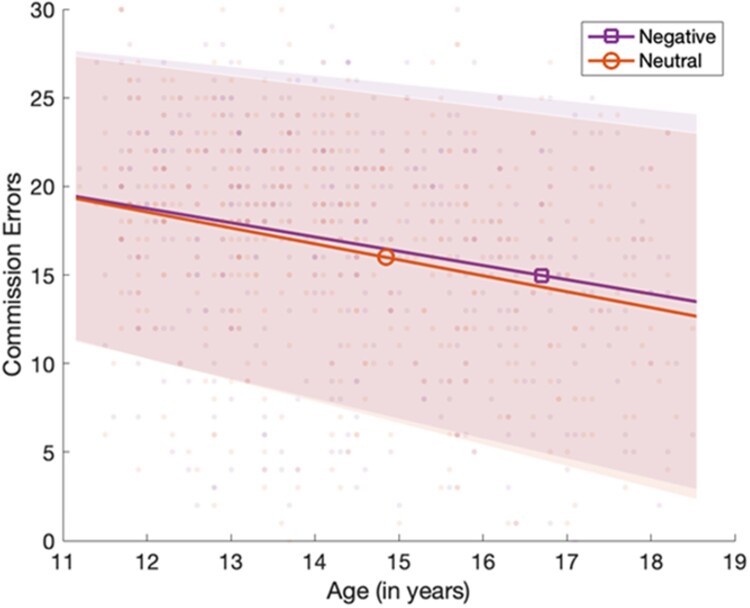
Mean numbers of aSART commission errors (with one standard error mean) in negative and neutral conditions showing reduced errors across both conditions with older age, from 11 to 18 years.

**Figure 3.**
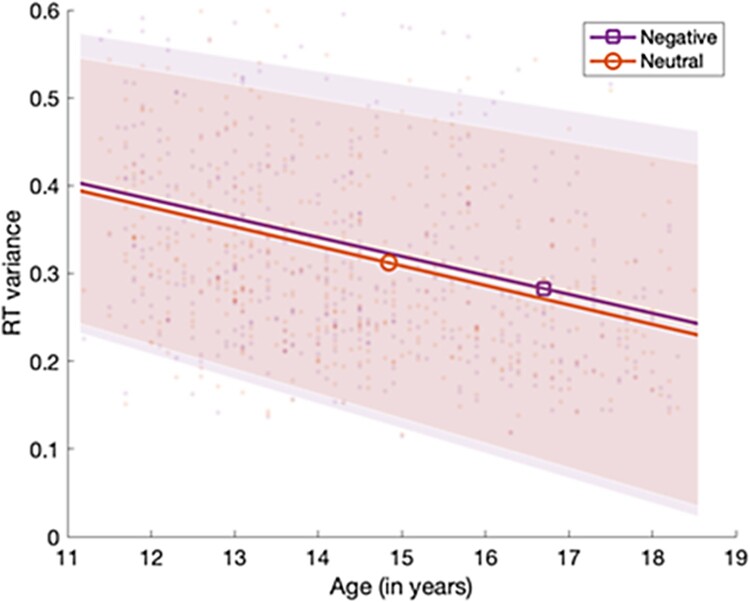
Mean aSART RT variance (with shading indicating one standard error of the mean mean) in negative and neutral conditions, showing reduced RT variance across both conditions with older age, across ages 11 to 18 years.

Failing to support our third hypothesis, that the influence of negative affective context would be greater for younger adolescents relative to their older peers, the difference in aSART performance between negative and neutral conditions appeared consistent across adolescence, when adjusting for gender and depression and using our computed commission errors index ($R_{\mathrm{adjusted}}^{2}$ = −.002, *F* (3, 440) = .69, *p *= .56, *β* = .05, *p *= .31), and computed RT variance index ($R_{\mathrm{adjusted}}^{2}$ = .01, *F* (3, 440) = 2.08, *p *= .10, *β* = .02, *p *= .66). A graph showing commission errors can be seen in Figure 2, and another for RT variance can be seen in Figure 3.

### aSART performance and elevated symptoms of depression and anxiety

#### Hypothesis four

To evaluate depression risk (hypothesis 4a), participants were allocated to two groups based on the established cut-off for the depression measure used, with scores of 16 and over indicating a risk of depression and scores of less than 16 indicating lower risk. Two-hundred and seventeen participants (161 Females, 56 Males; 38.6% of all participants) met the criteria for being “at risk”. Demographic data for the two groups are presented in Table 3.

**Table 3.** Means and standard deviations for age, IQ and depression for those characterised as at risk and at lower risk of depression.

	Lower risk (*n *= 229; Females *n *= 132)		At risk (*n *= 217; Females *n *= 161)		
	*M*	*SD*	*M*	*SD*	*p*
Age (years)	14.16	1.71	14.71	1.89	<.01*
IQ (standard score)	113.09	17.36	113.39	16.74	.85
Depression (raw score)	9.49	3.90	25.61	8.74	<.01*
Anxiety (raw score)	9.36	6.20	16.76	7.79	<.01*

There was a significant difference in the gender ratios between the “at risk” and “lower risk” groups, with a larger proportion of females in the “at risk” group (*X*^2^ (1, 446) = 13.54, *p* < .001). Also, there was also a significant difference in age between the risk groups, with the “at risk” group being older than the lower risk group (*t* (440) = 3.21, *p *= .001, *d *= .31, *CI* = 0.21–0.89). To adjust for these group differences, age and gender were covaried in subsequent analyses. There was no significant estimated IQ difference between groups. Finally, self-reported anxiety was higher in the at risk of depression group (*t* (440) = 11.08, *p *< .001, *d *= 1.05, *CI* = 6.09–8.71).

We conducted a series of separate linear regressions to examine the relationship between depression risk and aSART performance. For hypothesis 4a, because gender and age correlated with depression risk status, we included these in the model in block 1, with group (“at risk”, “lower risk”) in block 2, and aSART outcomes collated across task conditions (commission errors or RT variance) entered as the dependent variable. There were significant main effects of group with the “at risk” group making more commission errors than the lower risk group ($R_{\mathrm{adjusted}}^{2}$ = .099, *F* (3, 440) = 17.18, *p *< .001, *β *=* *−.15, *p* = .001). There was no significant difference between groups for RT variance ($R_{\mathrm{adjusted}}^{2}$ = .10, *F* (3, 440) = 17.96, *p *< .001, *β *=* *−.03, *p* = .53).

For anxiety (hypothesis 4b), we used linear regressions to examine the relationship between anxiety scores as a continuous variable and the aSART. Because gender correlated with anxiety score, we included this in the model in block 1, anxiety total score was entered in block 2 and aSART measure (commission errors or RT variance) was entered as the dependent variable. No significant differences were found for commission errors ($R_{\mathrm{adjusted}}^{2}$ = .04, *F* (2, 440) = 9.24, *p* < .01, *β* = .05, *p* = .26) or RT variance ($R_{\mathrm{adjusted}}^{2}$ = .01, *F* (2, 440) = 3.66, *p* = .03, *β* = −.08, *p* = .10).

#### Hypothesis five

To examine if there was a differential effect of aSART affective context as a function of depression risk (hypothesis 5a) we used the model from hypothesis 4a but this time with our computed commission errors or RT variance indices as the dependent variables. There were no significant effects for either commission errors ($R_{\mathrm{adjusted}}^{2}$ = 0.004, *F* (3, 440) = .38, *p* = .77, *β* = .003, *p* = .73) or RT variance ($R_{\mathrm{adjusted}}^{2}$ = .12, *F* (3, 440) = 1.38, *p* = .25, *β* = −.08, *p* = .08).

For anxiety (hypothesis 5b) similar linear regressions as for hypothesis 4b were conducted but now our computed aSART indices, for commission errors and RT variance, were entered as the dependent variables. These showed that anxiety score significantly predicted the difference between aSART conditions for RT variance ($R_{\mathrm{adjusted}}^{2}$ = .01, *F* (2, 440) = 3.11, *p* = .045, *β* = −.11, *p* = .03), such that those with higher anxiety scores showed less RT variance in the negative relative to the neutral condition than those with lower anxiety scores. There was no significant effect of aSART condition for commission errors ($R_{\mathrm{adjusted}}^{2}$ = −.003, *F* (2, 440) = .29, *p* = .75, *β* = −.31, *p* = .53).

#### Hypothesis six

To examine whether this differential pattern of the impact of negative contexts on aSART performance across those “at risk” versus those at lower risk for depression (hypothesis 6a), varied with age we conducted hierarchical regressions with gender, age, and depression risk (dummy variable coded 0/1) entered on step 1 and the interaction term of age x depression risk on step 2, with our computed commission errors index or RT variance index entered as the dependent variable. All results were non-significant (commission errors: $R_{\mathrm{adjusted}}^{2}$ = −.01, *F* (4, 435) = .276, *p* = .89, *β* = −.01, *p* = .98; RT variance: $R_{\mathrm{adjusted}}^{2}$ = .002, *F* (4, 435) = 1.24, *p* = .29, *β* = .21, *p* = .60).

For anxiety (hypothesis 6b), comparable hierarchical regressions were used with gender and anxiety score entered in Step 1, age on Step 2 and the commission errors or RT variance indices entered as the dependent variable. These too were non-significant (commission errors: $R_{\mathrm{adjusted}}^{2}$ = −.004, *F* (3, 431) = .45, *p* = .72, *β* = .04, *p* = .37; RT variance: $R_{\mathrm{adjusted}}^{2}$ = −.01, *F* (3, 431) = 2.05, *p* = .11, *β* = .001, *p* = .99).

## Discussion

This study investigated the effects of affective contexts on sustained attention during adolescence, age-related effects during that developmental period, and whether effects were modulated by depression risk and level of anxiety. To this end, we used an adapted affective version of the Sustained Attention to Response Task (SART) – the Affective SART (aSART) – in which participants were required to respond to frequent targets and withhold responses to infrequent targets while listening to either a negative or neutral background soundscape.

Our first hypothesis was that, across all participants, there would be a decrement in sustained attention performance in negatively valenced compared to neutral contexts. Our findings support this, with participants making significantly more commission errors and showing more RT variance in the negative contexts. This demonstrates that adolescents, like adults, have a relative difficulty in sustaining attention against a backdrop of aversive socio-affective stimuli. Of course, we cannot establish in this study whether this reduction in performance differs from that of adults. Another issue, to which we return to below, is whether these task-irrelevant sound sequences had differential effects *because* of their negative associations *per se*, or because of their *salience,* that may be in part, driven by their negative associations. These two sorts of interpretation can be found in the existing literature. It has been argued that negative stimuli (and/or the endogenous content/processes that they may trigger) are more effortful and elaborate to process than neutral stimuli (e.g. Gibb et al., 2016). Others have argued that negative stimuli are more likely to shift attentional resources away from task demands (i.e. compete through salience; Pratto & John, 1991). Effect sizes shown in this study were trivial to small. but this is entirely consistent with the existing literature of the effect of affective context on cognitive task performance (Schweizer et al., 2019). Even very small effects such as these are likely to have a clear impact on everyday cognition (Funder & Ozer, 2019) once one considers how frequently we might deploy sustained attention in affective contexts when managing daily life situations.

In line with our second hypothesis, the study also showed that the age-related performance improvements present in typical sustained attention tasks were also present in the aSART. Younger participants made more commission errors and showed more variance in their RTs than their older peers. However, as our study was cross-sectional this pattern of results needs to be replicated in a longitudinal study. A previous large-scale cross-sectional study of sustained attention showed a similar pattern of developmental change, with rapid performance improvements in error rate from early to mid-adolescence that were accompanied by a reduction in RT variance (Fortenbaugh et al., 2015).

We also predicted that the performance of younger participants would be more impacted by affective context than it would be in older adolescents. However, we found no support for such disproportionate influence. This suggests that the impact of affective context on sustained attention is consistent across adolescent development and therefore likely dependent on different processes to those underpinning the gradual improvement of SART performance overall across the adolescent years. This contrasts with studies that mostly show age-related reductions in interference from affective stimuli on task performance in other domains such as executive control. Previous studies have typically compared performance on these tasks with a broader age-range of participants and it may be that a greater age range in the present study would have revealed differences. Other studies have also typically used visual rather than auditory affective stimuli (see Schweizer et al., 2020 for a review) and it may be that any effects are modality-specific. It is also possible that the affective contexts used in the aSART, although sensitive enough to show general decrements in sustained attention performance, lack the sensitivity to identify differences related to age.

Our final hypotheses were concerned with whether the aSART revealed different cognitive and age-related profiles in relation to risk of depression and level of anxiety. We hypothesised that adolescents at risk of depression, and/or higher in ratings of anxiety, would show a decrement in overall performance on the aSART relative to a group that were at lower risk/less anxious. For depression, this was observed for commission errors but not for RT variability. Depressed adults, and to a lesser extent, depressed adolescents (e.g. Sommerfeldt et al., 2016), have been shown to make more errors on sustained attention tasks than the non-depressed. To our knowledge this is the first time that a group *at risk of depression* in any age group have also shown this deficit, suggesting that even relatively low levels of depressive symptomology are enough to make the sufferer more susceptible to lapses in sustained concentration. For level of anxiety, there were no significant differences in either commission errors or RT variance.

We also hypothesised that there would be a differential performance impact of the negative vs neutral context between the “at risk” and lower risk depression groups and/or as a function of level of anxiety. This non-directional hypothesis emerged from the equivocal findings associated with depression risk and anxiety level in studies examining other aspects of attention and across other cognitive domains (e.g. Aylward et al., 2017). Although, we did not observe a differential effect between negative and neutral contexts for depression risk for either commission errors or RT variance, we did find a small, but significant difference related to level of anxiety, with those with higher anxiety scores showing less RT variance in the negative context relative to the neutral context, compared to those with lower anxiety scores. It may be, as other studies have suggested, that anxious adolescents are better at attentional avoidance of potentially distressing information and this leads to better attentional performance in negative contexts (see Lisk et al., 2020 for a review). Similarly, it is also possible that negative contexts (relative to neutral ones) sharpen sustained attention in adolescents with higher anxiety compared to their lower risk peers due to the moderate levels of emotional arousal that they induce. Indeed, arousal states for sustained attention tasks have been shown to have a U-shaped function (The Yerkes-Dodson Law; Broadhurst, 1957). Low arousal states can lead to low task engagement and high arousal states to increased distractibility, both of which can negatively impact performance. By this analysis, it is possible then that the negative contexts in the aSART were enough to selectively raise arousal in the more anxious participants to an extent that their responses were not detrimentally impacted by the negative context. However, the difference between conditions was small, so this finding should be treated with caution. There were no significant effects of valence for commission errors as a function of anxiety level. Also, there was no significant differential pattern of results related to valence as a function of age for either depression risk or anxiety level.

It is useful to return to the issue of valence, salience and arousal at this stage. Are the effects that we are observing in terms of overall reductions in performance in the negative condition, and differential effects of the negative condition as a function of level of anxiety, related to negative valence *per se*, or perhaps to the greater distracting *salience* or *arousing properties* of these sounds (if indeed these can be meaningfully decoupled)? In their original development of the IADS, Bradley and Lang (2007) asked college students to rate sounds on the dimensions of arousal and valence. As with similar exercises with pictures, items with the highest and lowest ratings for pleasure tended to also have the highest ratings for arousal. It was also the case that a higher proportion of negative sounds received high arousal ratings than the positive, although this could be a consequence of their original sound selection. On some non-subjective measures of arousal, negative sounds had significantly greater effects (startle responses, facial muscle changes and heart rate deceleration) than positive, while on other indices (electrodermal response and recall performance) affective sounds in general (i.e. pleasant and unpleasant) had similarly increased effects when compared with neutral sounds. While there may be other acoustic features of interest, these differences were not related to the low-level property of sound intensity; in other words, affective stimuli were arousing/salient largely *because* of their affective association, and this was generally greater for the negative sounds. An interesting question remains as to whether task-irrelevant positive sounds of equal salience/arousing properties would have similar effects.

### Limitations and suggestions for future work

First, although data on a large number of participants was collected for this study, data collection was conducted in small groups, rather than individually. Although testing was conducted under exam conditions, and participants wore over-ear headphones, testing adolescents in groups could be negatively impacted by peer influence, particularly with regards to self-regulation (King et al., 2018) an essential element of sustained attention tasks.

Second, we chose to compare negative sounds to neutral sounds as we were particularly interested in the effect that negative stimuli have on sustained attention. Comparing negative sounds with neutral rather than positive sounds, allows us to better isolate the effect of negative sounds on sustained attention. However, we could have additionally included positive sounds that would have allowed us to establish if the decrements in performance observed were a result of negative sounds per se rather than a general distracting effect of affective stimuli. Relatedly, the negative sounds we included were chosen due to their valence and arousal properties rather than to the nature of the sounds themselves. For example, most of the sounds chosen related to threat (e.g. screaming, dog growling, car crash), while other were related to categories like disgust (e.g. vomiting, belching). Future studies might like to focus on threat or disgust, for example, to examine the individual effect of these on sustained attention performance due to threat vigilance processes.

## Conclusion

We used an adapted affective version of the SART to investigate the effects of a negative affective context (negative auditory stimuli) on sustained attention performance, across adolescence. When considering overall performance on the task (irrespective of task condition), performance improved as expected as a function of age. Importantly, although it is well established that deficits in sustained attention are present in those with depression, we also found that performance was worse in those adolescents deemed *at risk* of depression. In terms of differential aSART performance as a function of task context, across the sample as whole, sustained attention was poorer in the negative relative to the neutral context, with effect sizes comparable to those found in the adult literature (Schweizer et al., 2019). However, the extent of this decrement appeared invariant across adolescent development, despite an age-related improvement in SART performance overall. This suggests that age-related versus affective-context related decrements in performance may be underpinned by different mechanisms. We further found that this decrement in performance associated with negative affective contexts was eliminated in adolescents with higher levels of anxiety. This suggests that the moderate levels of emotional arousal, and/or enhanced levels of attentional avoidance to potentially distressing information, associated with moderate mental health symptomatology may help mitigate the expected deleterious effects of negative stimuli on sustained attention in these vulnerable adolescents. Taken together, these results suggest that the aSART is a criterion-valid measure of sustained attention in affective contexts that might be useful in identifying a marker of cognitive resilience in adolescents higher in level of anxiety.

## Supplementary Material

Supplementary_material-_sustaining_attention_in_affective_contexts_R1 new.docx
