# Supplementary material for: Sustaining attention in affective contexts during adolescence: age-related differences and association with elevated symptoms of depression and anxiety
Source: Cogn Emot. 2024 May 7;38(7):1122–34. doi: 10.1080/02699931.2024.2348730 (PMC11573249; doi:10.1080/02699931.2024.2348730)
Supplement: Supplementary_material-_sustaining_attention_in_affective_contexts_R1 new.docx [file PCEM_A_2348730_SM6400.docx]

**SUPPLEMENTAL MATERIALS**

**Sustaining attention in affective contexts during adolescence: Age-related differences and association with mental health problems**

Dunning, D. L.^1,2^, Parker, J.^1^, Griffiths, K.^1^, Bennett, M.^1^, Archer-Boyd, A.^1^, Bevan, A.^1^, Ahmed, S.^3^, Griffin, C.^3^, Foulkes, L.^4^, Leung, J.T.^3^, Sakhardande, A.^3^, Manly, T. ^1^, Kuyken, W.^5^, Williams, J.M.G.^5^, Blakemore, S-J.^3,6^ & Dalgleish, T.^1,7^

Author affiliations:

^1^ Medical Research Council Cognition and Brain Sciences Unit, Cambridge University, Cambridge, CB2 7EF, UK

^2^University of Hertfordshire, Hatfield, Hertfordshire, AL10 9AB

^3^ Institute of Cognitive Neuroscience, University College London, London, WC1N 3AR, UK

^4^ School of Psychology and Language Sciences, University College London, WC1H 0AP, UK

^5^Department of Psychiatry, University of Oxford, Oxford, OX3 7JX, UK

^6^Department of Psychology, Cambridge University, Cambridge, CB3 3EB, UK

^7^Cambridgeshire and Peterborough NHS Foundation Trust, Cambridge, UK

Corresponding authors: [d.dunning@herts.ac.uk](mailto:d.dunning@herts.ac.uk) and [tim.dalgleish@mrc-cbu.cam.ac.uk](mailto:tim.dalgleish@mrc-cbu.cam.ac.uk)

**Contents**

**Method Page**

Participants……………………………………………………………………………..……3

Additional information on measures………….…………………..…….……..3

Affective Sustained Attention to Response Test (aSART)……….……..3

Instructions…………………………………………………………….……….5

aSART soundfile ratings (table S1)……………………………..…….7

Center for Epidemiologic Studies Depression Scale (CES-D)………... 9

Revised Children's Anxiety and Depression Scale (RCADS)……..…. 10

Cattell Culture Fair Intelligence Test (CCFIT)…………………………..…..10

**Results** (reaction time and Omission errors)…………………………..………………11

Hypothesis 1……………………………………………………………..………………. 11

Table S2……………………………………………………….…………………12

Hypothesis 2…………………………………………..…………………………………. 12

Hypothesis 3……………………………………………………..………………………. 12

Hypothesis 4……………………………………………………..………………………. 13

Table S3………………………………………………………………………… 13

Hypothesis 5………………………………………………………..……………………. 14

Hypothesis 6……………………………………………..………………………………. 14

**References**……………………………………………………………….…….……………………….15

**Method**

Participants

Four hundred and eighty-five participants (320 females) aged 11-18 years (M = 14.40, SD = 1.80) were recruited from 15 schools and colleges in Greater London and Cambridge (U.K.). All students aged 11-18 years from these schools were given an information sheet with details about the study and an opt-in consent form. All students had the opportunity to take part in the study unless: (i) they had a self-reported diagnosis of a learning difficulty or a neurodevelopmental or neurological disorder; (ii) they had a self-reported mood disorder. After signed consent forms were returned, participants were selected on a first-come first-served basis until our target sample size was reached. The study was approved by the University of Cambridge and University College London Research Ethics Committees. Participants aged under 16 needed parental consent and those aged 16-18 provided their own consent. Assent was obtained from all participants. Participants were compensated with £15 in shopping vouchers for taking part in the research, which took place in small groups at the participants’ school or college.

Schools and colleges were chosen from those that responded to recruitment emails as part of a larger project investigating the mechanisms of mindfulness training in adolescence (Dalgleish, et al., 2020; Dunning, et al., 2022). All testing took place at baseline assessment, so before any mindfulness training took place.

Additional information on measures

*Affective Sustained Attention to Response Test (aSART).*

The original version of the SART was designed as a simple, controlled, reliable and valid measure of lapses in sustained attention. The aSART adapts the original SART through the introduction of different auditory background stimuli – affective versus neutral – to evaluate whether attentional lapses vary as a function of affective context. Apart from the addition of background stimuli, the aSART was identical to the original SART. Both were computer-administered tasks that involved the withholding of key presses to rare (one in nine) targets presented on the screen. Specifically, targets were drawn from the numbers 1-9 and were presented one digit at a time. The participant was simply asked to respond to the appearance of each digit by pressing the space bar (‘Go’ trials). The exception to this was when the number ‘3’ appeared, to which no response should be made (‘No-go’ trials). For the aSART, the response window was 1150 milliseconds (ms) - each digit was on screen for 400 ms, followed by a mask (a fixation cross) for 750 ms. Five-hundred and forty trials were presented, 60 of which were no-go trials, over a period of 12 mins.

While completing the task, in a within-subjects design, participants listened to a continuous background stream of either neutral- or negative-valence sounds through headphones. The 540 trials were divided into six blocks of 90 trials each. In three of the blocks, participants heard a stream of negative sounds (e.g., an alarm clock going off) and in the other three blocks they heard a stream of affectively neutral sounds (e.g., crowd murmur). The six blocks were randomly presented. The sounds were taken from the International Affective Digitized Sounds (IADS) corpus (a library of sounds pre-rated for valence and arousal by college attending adults; Bradley & Lang, 2007). Each sound file lasted approx. 6 seconds. The files were concatenated using a custom script written in MATLAB 2014a (Mathworks, 2014). Because the sound files (.wavs) were recorded at various sample rates (8 to 44.1 kHz) they were first resampled to 44.1 kHz in MATLAB to standardise presentation. A list of all sounds used, along with the adult and adolescent valence ratings form an unreported pilot study, can be found in the Supplementary materials (Table S1).

Outcomes measures were as follows: Commission errors were the total number of space bar responses that occurred following the presentation of the no-go digit, 3. Omission errors were the total number of go trials to which a no response was made before the onset of the next trial (1150 ms). Correct RT was the mean interval (ms) between digit onset and response on go trials. RT variance was calculated separately for each participant by dividing the standard deviation associated with their mean correct RT by their correct RT (standard deviations of reaction times are generally proportionate to the overall magnitude of the mean RT, this *coefficient of variation* approach effectively removes the influence of overall RT allowing clearer comparison of differences specifically in variability).

As noted, the key outcome variables for the present study were commission errors and RT variance. In addition, we computed indices to measure the effect of affective context by subtracting scores on the key aSART outcome variables in the neutral condition from scores in the negative condition, such that larger scores represented a bigger influence of affective context.

Instructions for the aSART

Before commencing the aSART, participants were asked to put on a set of over-ear headphones and given the following series of instructions and practice trials. The first set of instructions read: *In this task you will see numbers between 1 and 9 appear on the screen in a random sequence. After you see each number press the space bar.* These instructions were followed by nine practice trials (the numbers 1-9 in a random order). After this, a second set of instructions appeared that read: *Well done. Now, there is one rule to remember: you should press the space bar after each number EXCEPT 3. If you see a 3, do not press the space bar – just wait for the next number to appear.* This was followed by 18 practice trials (the numbers 1-9 repeated twice in random order). After this, the third set of instructions appeared: *Good. There is one last thing to be aware of. While you are doing the task you will hear some sounds over your headphones. These may be funny, strange or even a little scary. It is important that you try and ignore these sounds and concentrate on the task. Remember press the space bar for every number EXCEPT 3. If you see a 3, do not press the space bar – just wait for the next number to appear.* This was followed by 18 final practice trials (the numbers 1-9 repeated twice in a random order), accompanied by a range of neutral sounds. Finally, just before starting the task proper participants were informed: *This experiment will last about 12 minutes. It is really important that you concentrate for the whole time and do not miss any of the numbers. Could you hear the sounds? Is everything clear? It is important that you ask the researcher now if you have any questions as once you have started you won't be able to pause the experiment.*

aSART soundfile ratings

**Table S1**

*Rating of all negative and neutral sounds used in the aSART*

|  |  | Adolescent ratings | | | | IADS ratings | | | |
| --- | --- | --- | --- | --- | --- | --- | --- | --- | --- |
| **IADS Soundfile number** | **IADS Description** | **Pleasure mean** | **sd** | **Arousal mean** | **sd** | **Pleasure mean** | **sd** | **Arousal mean** | **sd** |
| **Negative sounds** |  |  |  |  |  |  |  |  |  |
| 102 | Cat | 2.33 | 2.550 | 5.667 | 1.73 | 4.63 | 2.17 | 4.91 | 1.97 |
| 105 | puppy | 2.11 | 0.928 | 6.556 | 1.59 | 2.88 | 2.14 | 6.40 | 2.13 |
| 106 | growl1 | 3.33 | 1.118 | 5.889 | 1.17 | 3.37 | 1.64 | 6.39 | 1.62 |
| 115 | Bees | 2.56 | 1.67 | 5.56 | 2.01 | 2.16 | 1.33 | 7.03 | 1.91 |
| 116 | Buzzing | 2.11 | 1.05 | 6.11 | 1.45 | 3.02 | 1.65 | 6.51 | 2.13 |
| 133 | Growl2 | 3.89 | 2.26 | 5.67 | 1.58 | 3.79 | 1.69 | 6.23 | 1.84 |
| 241 | MaleCough | 3.33 | 2.06 | 5.22 | 0.67 | 2.46 | 1.53 | 5.87 | 2.06 |
| 242 | femalecough | 3.22 | 0.97 | 5.56 | 1.59 | 2.80 | 1.86 | 5.39 | 1.91 |
| 244 | manwheeze | 2.67 | 0.87 | 6.00 | 1.00 | 2.44 | 1.34 | 6.31 | 1.85 |
| 255 | Vomit | 1.11 | 0.33 | 6.00 | 2.29 | 2.08 | 1.78 | 6.59 | 2.08 |
| 261 | babycry | 2.33 | 0.87 | 6.33 | 1.41 | 2.75 | 1.68 | 6.51 | 1.96 |
| 275 | scream | 2.33 | 1.58 | 6.89 | 1.69 | 2.05 | 1.62 | 8.16 | 2.15 |
| 276 | femscream2 | 1.22 | 0.67 | 7.11 | 1.76 | 1.93 | 1.63 | 7.77 | 1.50 |
| 277 | femscream3 | 1.44 | 0.88 | 7.11 | 1.54 | 1.63 | 1.13 | 7.79 | 1.63 |
| 280 | WomanCrying | 3.67 | 2.24 | 5.67 | 0.71 | 3.65 | 1.87 | 5.33 | 1.46 |
| 288 | creep | 1.89 | 1.05 | 6.22 | 1.30 | 2.71 | 1.75 | 6.82 | 1.63 |
| 289 | gunshot | 1.78 | 1.09 | 5.78 | 1.30 | 3.08 | 1.71 | 6.57 | 1.80 |
| 291 | prowler | 3.33 | 1.23 | 5.78 | 0.83 | 3.67 | 1.70 | 6.35 | 1.76 |
| 292 | malescream | 2.00 | 1.00 | 6.11 | 1.17 | 1.99 | 1.41 | 7.28 | 1.74 |
| 293 | mansobbing | 2.11 | 1.05 | 5.67 | 1.32 | 3.08 | 1.92 | 5.74 | 1.69 |
| 295 | couplesobbing | 2.33 | 1.12 | 5.56 | 1.33 | 3.27 | 2.39 | 5.79 | 1.81 |
| 296 | womencrying | 2.11 | 0.93 | 6.00 | 1.73 | 2.06 | 1.22 | 6.07 | 1.97 |
| 310 | crowd1 | 3.11 | 1.36 | 6.11 | 1.27 | 3.89 | 2.32 | 6.78 | 2.02 |
| 420 | carhorns | 2.56 | 1.24 | 6.11 | 1.27 | 2.34 | 1.51 | 7.08 | 2.06 |
| 422 | tireskids | 3.56 | 1.59 | 6.44 | 1.42 | 2.22 | 1.47 | 7.52 | 1.90 |
| 423 | injury | 3.33 | 1.73 | 5.78 | 1.39 | 3.31 | 1.79 | 6.23 | 1.60 |
| 424 | carwreck | 2.56 | 0.88 | 6.22 | 0.97 | 2.04 | 1.52 | 7.99 | 1.66 |
| 501 | planecrash | 2.78 | 1.56 | 6.00 | 1.41 | 2.74 | 1.76 | 6.93 | 1.91 |
| 502 | enginefailure | 3.44 | 1.13 | 5.56 | 1.13 | 3.15 | 2.01 | 6.32 | 1.87 |
| 624 | airraid | 3.44 | 1.13 | 6.22 | 1.39 | 2.82 | 1.75 | 7.10 | 2.10 |
| 625 | mayday | 2.78 | 1.56 | 6.89 | 1.17 | 3.35 | 2.03 | 6.94 | 1.77 |
| 626 | explosion | 2.56 | 1.59 | 6.56 | 1.74 | 3.37 | 1.98 | 6.61 | 1.71 |
| 699 | bomb | 2.78 | 1.09 | 6.22 | 1.56 | 3.59 | 2.07 | 6.15 | 2.36 |
| 702 | belch | 3.78 | 2.17 | 5.56 | 0.88 | 4.45 | 2.57 | 5.37 | 1.95 |

**Table S1 (continued)**

|  |  | Adolescent ratings | | | | IADS ratings | | | |
| --- | --- | --- | --- | --- | --- | --- | --- | --- | --- |
| **IADS Soundfile number** | **IADS Description** | **Pleasure mean** | **sd** | **Arousal mean** | **sd** | **Pleasure mean** | **sd** | **Arousal mean** | **sd** |
| **Negative sounds** |  |  |  |  |  |  |  |  |  |
| 703 | BusySignal | 3.44 | 2.01 | 5.44 | 1.59 | 2.65 | 1.59 | 5.68 | 1.89 |
| 704 | phone1 | 3.89 | 1.54 | 5.56 | 1.33 | 5.49 | 1.98 | 6.54 | 2.17 |
| 706 | war | 3.00 | 1.41 | 6.00 | 1.32 | 4.16 | 1.68 | 5.30 | 1.83 |
| 709 | alarmclock | 3.33 | 1.58 | 6.78 | 1.79 | 2.78 | 1.93 | 7.54 | 2.28 |
| 710 | cuckoo | 3.89 | 1.45 | 6.67 | 1.66 | 4.27 | 2.04 | 6.24 | 1.88 |
| 711 | siren1 | 3.00 | 1.00 | 7.33 | 0.87 | 2.61 | 1.59 | 7.39 | 2.02 |
| 712 | buzzer | 2.67 | 1.32 | 7.22 | 1.30 | 2.42 | 1.62 | 7.98 | 1.99 |
| 713 | sirens | 3.56 | 1.24 | 5.89 | 1.45 | 2.95 | 1.71 | 6.98 | 1.53 |
| 714 | siren2 | 3.00 | 1.12 | 6.78 | 1.56 | 3.10 | 1.67 | 6.94 | 1.85 |
| 715 | alarm | 3.00 | 1.12 | 6.78 | 1.39 | 4.30 | 2.50 | 6.99 | 1.79 |
| 730 | glassbreak | 2.67 | 1.58 | 5.78 | 0.83 | 3.22 | 1.45 | 6.23 | 1.78 |
| 732 | Crash | 2.00 | 0.87 | 6.78 | 1.64 | 2.89 | 1.68 | 6.98 | 1.75 |
| **Neutral sounds** |  |  |  |  |  |  |  |  |  |
| 113 | Cows | 5.67 | 2.00 | 4.89 | 1.54 | 5.45 | 1.71 | 4.88 | 1.95 |
| 130 | pig | 5.67 | 1.66 | 5.00 | 1.41 | 4.64 | 2.11 | 4.93 | 1.98 |
| 132 | chickens | 5.22 | 1.20 | 5.33 | 1.32 | 5.64 | 1.76 | 4.77 | 1.73 |
| 152 | tropical | 5.67 | 1.73 | 4.33 | 1.41 | 5.23 | 2.28 | 5.51 | 2.23 |
| 170 | night | 5.67 | 0.87 | 4.67 | 1.50 | 5.31 | 2.12 | 4.60 | 2.07 |
| 171 | countrynight | 5.56 | 1.59 | 4.00 | 1.73 | 5.59 | 1.79 | 3.71 | 2.05 |
| 172 | Brook | 5.89 | 2.52 | 4.22 | 2.28 | 6.62 | 1.69 | 3.36 | 2.07 |
| 224 | kids2 | 5.44 | 1.67 | 5.89 | 1.62 | 6.11 | 1.90 | 5.64 | 1.89 |
| 245 | hiccup | 4.56 | 1.51 | 5.22 | 1.79 | 4.18 | 1.85 | 5.05 | 1.82 |
| 246 | heartbeat | 4.22 | 1.09 | 5.00 | 0.50 | 4.83 | 1.81 | 4.65 | 2.49 |
| 250 | malesneeze | 4.33 | 1.41 | 5.00 | 0.87 | 3.54 | 1.57 | 4.94 | 1.90 |
| 254 | videogame | 5.78 | 1.56 | 5.22 | 1.30 | 6.17 | 1.65 | 5.58 | 1.99 |
| 262 | yawn | 5.67 | 1.50 | 4.00 | 0.87 | 5.26 | 1.58 | 2.88 | 1.74 |
| 270 | whistling | 5.33 | 1.80 | 4.78 | 1.64 | 6.10 | 1.83 | 4.23 | 2.06 |
| 320 | office1 | 5.00 | 1.32 | 5.78 | 0.83 | 4.23 | 1.56 | 5.48 | 1.95 |
| 358 | Writing | 5.00 | 2.00 | 5.22 | 1.20 | 4.52 | 1.34 | 4.87 | 1.98 |
| 364 | Bar | 5.56 | 2.07 | 5.89 | 1.54 | 5.19 | 1.85 | 5.62 | 1.75 |
| 373 | paint | 5.11 | 0.60 | 4.67 | 1.00 | 5.09 | 1.55 | 4.65 | 2.17 |
| 374 | Sink | 5.89 | 0.93 | 4.67 | 2.12 | 5.60 | 1.35 | 4.23 | 1.89 |
| 375 | polaroid | 5.78 | 1.72 | 4.33 | 1.94 | 5.99 | 1.60 | 4.48 | 1.74 |

**Table S1 (continued)**

|  |  | Adolescent ratings | | | | IADS ratings | | | |
| --- | --- | --- | --- | --- | --- | --- | --- | --- | --- |
| **IADS Soundfile number** | **IADS Description** | **Pleasure mean** | **sd** | **Arousal mean** | **sd** | **Pleasure mean** | **sd** | **Arousal mean** | **sd** |
| **Neutral sounds** |  |  |  |  |  |  |  |  |  |
| 376 | lawnmower | 5.11 | 0.78 | 5.00 | 1.32 | 4.88 | 1.62 | 4.60 | 1.93 |
| 377 | Rain1 | 5.44 | 1.74 | 4.11 | 2.21 | 5.84 | 1.73 | 3.93 | 1.87 |
| 378 | doorbell | 5.11 | 1.17 | 5.67 | 1.00 | 6.06 | 2.01 | 6.15 | 2.22 |
| 382 | shovel | 4.67 | 1.66 | 4.89 | 0.33 | 4.33 | 1.42 | 4.64 | 1.88 |
| 403 | helicopter1 | 4.67 | 1.58 | 5.33 | 1.12 | 5.57 | 1.83 | 5.56 | 1.99 |
| 410 | helicopter2 | 4.78 | 0.67 | 5.00 | 0.87 | 4.86 | 1.48 | 5.89 | 2.06 |
| 425 | train | 5.22 | 1.09 | 5.56 | 1.01 | 5.09 | 1.42 | 5.15 | 1.54 |
| 500 | Wind | 4.22 | 2.11 | 5.00 | 1.58 | 4.32 | 2.03 | 5.40 | 1.93 |
| 602 | Thunderstorm | 5.33 | 1.94 | 5.44 | 2.40 | 5.99 | 2.23 | 3.77 | 1.74 |
| 627 | rain1 | 4.11 | 1.17 | 5.00 | 0.87 | 4.83 | 1.89 | 4.65 | 2.12 |
| 698 | rain2 | 5.67 | 1.73 | 5.33 | 1.94 | 5.18 | 1.94 | 4.12 | 1.98 |
| 700 | toilet | 4.78 | 0.67 | 4.89 | 1.54 | 4.68 | 1.61 | 4.03 | 2.36 |
| 701 | fan | 4.67 | 0.72 | 5.00 | 0.87 | 4.95 | 1.47 | 4.41 | 2.06 |
| 705 | phone2 | 5.11 | 0.33 | 4.67 | 0.71 | 5.35 | 1.43 | 4.15 | 1.72 |
| 708 | clock | 4.78 | 1.20 | 5.22 | 1.64 | 4.34 | 1.42 | 3.51 | 2.05 |
| 720 | brushteeth | 5.11 | 1.45 | 4.89 | 1.69 | 4.86 | 1.80 | 4.18 | 1.79 |
| 721 | beer | 5.22 | 1.72 | 5.33 | 1.32 | 6.71 | 1.75 | 5.00 | 2.12 |
| 722 | Walking | 4.44 | 0.73 | 5.22 | 2.05 | 4.83 | 1.22 | 4.97 | 1.82 |
| 723 | Radio | 4.33 | 2.00 | 5.44 | 1.88 | 5.34 | 1.97 | 4.91 | 1.74 |
| 725 | SodaFizz | 5.11 | 2.09 | 4.78 | 2.59 | 6.61 | 1.80 | 4.55 | 2.17 |
| 726 | corkpour | 5.33 | 1.50 | 5.56 | 1.24 | 6.82 | 1.60 | 4.51 | 2.08 |
| 728 | paper1 | 5.22 | 1.48 | 5.67 | 1.66 | 4.72 | 1.26 | 4.35 | 2.09 |
| 729 | paper2 | 4.78 | 1.20 | 4.44 | 1.81 | 4.30 | 1.69 | 5.79 | 1.90 |

*Center for Epidemiologic Studies Depression Scale (CES-D)*

The CES-D (Radloff, 1977, 1991) is a 20-item self-report measure in which participants were asked to rate how often over the past week they had experienced symptoms associated with depression (e.g. *I felt that everything I did was an effort*). Responses range from 0 to 3 (0 = Rarely or none of the time; 1 = Some or little of the time; 2 = Moderately or much of the time; 3 = Most or almost all of the time. Four items are reverse scored (e.g. *I felt hopeful about the future*). Responses are summed and scores ranged from 0 to 60, with higher scores indicating greater depressive symptoms. The clinical cut-off for being at risk of depression is a score of 16. The re-test reliability of the CES-D for is α=.85 (Radloff, 1991).

*Revised Children's Anxiety and Depression Scale (RCADS)*

The RCADS (Chorpita, Yim, Moffitt, Umemoto & Francis, 2000) is a 15-item self-report questionnaire that measures symptoms of anxiety and low mood (e.g. I worry that I will suddenly get a scared feeling when there is nothing to be afraid of). Participants record on a four-point scale the frequency of their symptoms (0= Never; 1= Sometimes; 2=Often; 3=Always). The items in the RCADS are summed to give a total score ranging from 0-40 with greater scores indicating greater symptoms of anxiety. The re-test reliability of the RCADS for is α=.95 (Kösters, et al., 2000).

*Cattell Culture Fair Intelligence Test (CCFIT)*

The CCFIT (Cattell, 1963) is a measure of non-verbal intelligence that minimises sociocultural and environmental influences. A paper and pencil version of Scale 2 Form A of the test was used. This comprised four timed subtests that consisted of questions involving the relationships between pictures of abstract geometric shapes (e.g., completing a sequence of pictures of shapes or choosing a shape that is different from others). Correct responses were summed, and age-appropriate standard scores were calculated based on a set of existing norms. The internal reliability of the CCFIT is α=.77 (Nenty & Dinero, 1981).

**Results for reaction time (RT) and omission errors**

Hypotheses

(1) That performance would be poorer in the negative aSART condition versus the neutral condition, across the sample as a whole.

(2) That overall performance on the aSART (irrespective of the valence of the task condition) would be associated with older age across adolescence.

(3) That the influence of affective context, i.e. a relative decrement in aSART performance in the negative versus neutral condition, would be greater for younger adolescents relative to their older peers.

(4) Adolescents deemed to be at risk for depression according to cut-offs on a measure of depression or with elevated symptoms of anxiety, would show *overall* worse performance on the aSART relative to those deemed to be lower risk of depression or with lower symptoms of anxiety.

(5) Based on the extant literature, we had a non-directional hypothesis that there would be a differential effect of affective context, i.e. a relative difference in aSART performance in affective versus neutral conditions, in those adolescents deemed at risk of depression, compared with their lower risk peers, and in those with higher levels of anxiety, compared to those with lower levels.

(6) That any differential effect of affective context in those at risk of depression and higher in ratings of anxiety would be greater in younger, relative to older, adolescents.

aSART performance

Hypothesis 1- performance would be poorer in the negative aSART condition versus the neutral, across the sample as a whole.

To test our first hypothesis that aSART performance would be poorer in negative relative to neutral contexts, we analysed within-participant differences between affective conditions using repeated-measures general linear models. However, there were no significant differences between negative and neutral conditions for either reaction time (RT) (F (1, 445) = 2.91, p = .09, d = .17), or omission errors (F(1,444) = .00, p = .99, d = .00). Table S2 shows the means, standard deviation and range for RT, omission errors, IQ, CES-D and RCADS.

**Table S2**

*Descriptives for omission errors, RT and anxiety*

|  | Mean (sd) | Range |
| --- | --- | --- |
| aSART |  |  |
| RT, negative condition (ms) | 352.20 (102.74) | 205.92 -704.15 |
| RT, neutral condition (ms) | 349.08 (103.08) | 207.49 - 697.03 |
| Omission errors, negative condition | 13.13 (10.15) | 0 - 58 |
| Omission errors, neutral condition | 13.12 (9.50) | 0 - 58 |
| RCADS Total | 12.96 (7.93) | 0 - 40 |

Note. aSART = affective Sustained Attention to Response Task; IQ = Cattell Culture Fair Intelligence Test; CES-D = Center for Epidemiological Studies-Depression Scale; RCADS = Revised Children's Anxiety and Depression Scale; ms = milliseconds; RT = reaction time

Hypothesis 2. *The developmental trajectory of aSART performance*

Linear regressions (adjusting for gender and depression) showed that older participants made fewer omission errors (R^2^ _adjusted_ =.09, F (3, 440) = 14.58, p = <.01, β = -.29, p < .01) than younger participants. There were no significant difference in RT as a function of age (R^2^ _adjusted_ =.02, F (3, 440) = 4.54, p = <.05, β = -.09, p = .08).

Hypothesis 3. The influence of affective context

The difference between aSART performance in the negative and neutral conditions appeared consistent across the adolescent developmental trajectory with no differential influence of age across affective conditions, adjusting for gender and depression, using our computed index (negative condition – neutral condition), RT Index= R^2^ _adjusted_ =.003, F(3, 440) = 1.42, p =. 24, β = -.09, p < .08; Omission Error index = R^2^ _adjusted_ = -.004, F (3, 440) = .40, p = .75, β = .032, p = .52.

Hypothesis 4. *aSART performance and mental health*

Participants were allocated to two groups based on CES-D scores, with scores of 19 and over indicating a risk of depression and scores less than 19 indicating lower risk. One-hundred and sixty-four participants (125 Females, 39 Males) met the criteria for ‘at risk’ (36.8% of all participants). Demographic and aSART data for Omission errors and RT for the two groups are presented in Table S3.

**Table S3**

*Descriptives for demographic and aSART data for those characterized as at risk and not at risk of depression*

|  | Not at Risk (n 282) | | At Risk (n 164) | |
| --- | --- | --- | --- | --- |
|  | M | sd | M | sd |
| Age | 14.19 | 1.76 | 14.80 | 1.81 |
| RCADS total | 9.928 | 6.24 | 18.09 | 7.87 |
| aSART measures |  |  |  |  |
| RT negative (ms) | 359.00 | 108.10 | 338.70 | 102.74 |
| RT neutral (ms) | 356.29 | 108.08 | 336.63 | 103.08 |
| Omission errors negative | 13.51 | 10.10 | 12.46 | 10.15 |
| Omission errors neutral | 13.63 | 9.78 | 12.25 | 9.50 |

Note. RCADS = Revised Children's Anxiety and Depression Scale; aSART = affective Sustained Attention to Response Task; ms = milliseconds; RT= reaction time

Mixed-model general linear models with aSART Condition (negative, neutral) as the within-subjects factor and Group (‘at risk’ ‘not at risk’) as the between-subjects factor, with age and gender included as covariates. We found no significant main effect of Group (all Fs <2, all ps > .05). There was also no Group by Condition interaction with those at risk of depression showing no difference in RT (F(1,437) = .04, p = .84, d = .02) or omission errors (F(1,437) = .12, p = .73, d = .03) than those not at risk.

The analysis involving depression risk status was corroborated using our continuous measure of anxiety. Linear regression (adjusting for gender) showed no difference in aSART performance as a function of anxiety, all Fs<2. Further Linear regression (adjusting for gender) showed that RCADS total did not significantly predicted a difference between conditions as measured by our aSART index. This was true for RT (R^2^ _adjusted_ =-.001, F(2, 440) = .75, p = .48, β= -.06, p = .23) and omissions errors (R^2^ _adjusted_ =-.004, F(2, 440) = .10, p = .90, β = -.01, p = .87).

Hypothesis five - *Affective context of a function of mental health risk*

To examine if there was a differential effect of aSART affective context as a function of depression risk (hypothesis 5a) we used the model from Hypothesis 4 but this time with our Computed Omission Errors and RT Indices as the dependent variables. There were no significant effects for either omission errors (R^2^ _adjusted_ =0.01, F (3, 440) =.15, p=.93, β= .003, p=.95) or RT (R^2^ _adjusted_ =.002, F (3, 440) =1.34, p=.26, β=.03, p=.57).

For anxiety similar linear regressions were conducted with gender included in the model in block 1, anxiety score in block 2 but now with our Computed aSART indices entered as the dependent variables. These showed that anxiety score did not significantly predict the difference between aSART conditions for RT (R^2^ _adjusted_ =.01, F (2, 440) =1.77, p=.15, β= -.06, p=.21) or Omission errors (R^2^ _adjusted_ =-.01, F (2, 440) =.15, p=.93, β= -.01, p=.89).

Hypothesis 6. *That any differential effect of affective context in those at risk of mental health problems would be greater in younger, relative to older, adolescents.*

To examine whether this differential pattern of the impact of negative contexts on aSART performance across those ‘at risk’ versus those at lower risk for depression, varied with age we conducted hierarchical regressions with gender, age, and depression risk (as a dummy variable coded 0/1) entered on Step 1 and the interaction term of age x depression risk on Step 2, with our computed Omission Errors Index or RT Index entered as the dependent variable. All results were non-significant (omission errors: R^2^ _adjusted_ = -.01, F (4, 440) = .232, p=.92, β= .28, p=.48; RT: R^2^ _adjusted_ = .004, F (4, 440) = 1.45, p=.22, β= .53, p=.19).

For anxiety, comparable hierarchical regressions were used with gender and anxiety score entered in Step 1, age on Step 2 and the computed Omission Errors Index or RT Index entered as the dependent variable. There was a non-significant result for omission errors (R^2^ _adjusted_ = -.01, F (3, 436) = .15, p=.93, β= .02, p=.62), and although there was a significant result for RT (R^2^ _adjusted_ = .01, F (3, 436) = 1.77, p=.15, β= .10, p=.05), the model was not significant.

**References**

Bradley, M. M. & Lang, P. J. (2007). The International Affective Digitized Sounds (2nd Edition; IADS-2): Affective ratings of sounds and instruction manual. Technical report B-3. University of Florida, Gainesville, Fl.

Cattell, R. B. (1963). Theory of fluid and crystallized intelligence: A critical experiment. *Journal of Educational Psychology, 54*, 1-22. [https://doi.org/10.1037/h0046743](https://psycnet.apa.org/doi/10.1037/h0046743)

Chorpita, B.F., Moffitt, C.E., & Gray, J. (2005). Psychometric properties of the Revised Child Anxiety and Depression Scale in a clinical sample. *Behaviour Research and Therapy, 43*, 309-322. <https://doi.org/10.1016/j.brat.2004.02.004>

Dalgleish, T., Blakemore, S.-J., Dunning, D. L., Ahmed, S., Leung, J. T., Foulkes, L., & Griffiths, K. (2020, December 9). The impact of mindfulness training in adolescence on socio-cognitive-affective processes and on mental health before and after COVID-19: A trial protocol and analysis plan. Retrieved from [osf.io/d6y9q](https://osf.io/d6y9q/)

Dunning, D.L., Ahmed, S., Foulkes, L., Griffin, C., Griffiths, K., Leung, J. T., ... & MYRIAD Team. (2022). The impact of mindfulness training in early adolescence on affective executive control, and on later mental health during the COVID-19 pandemic: a randomised controlled trial. *Evidence-based mental health*, *25*(3), 110-116. <http://dx.doi.org/10.1136/ebmental-2022-300460>

Kösters, M. P., Chinapaw, M. J., Zwaanswijk, M., van der Wal, M. F., & Koot, H. M. (2015). Structure, reliability, and validity of the revised child anxiety and depression scale (RCADS) in a multi-ethnic urban sample of Dutch children. *BMC psychiatry*, *15*(1), 132.

MATLAB and Statistics Toolbox Release 2014a, The MathWorks, Inc., Natick, Massachusetts, United States.

Nenty, H. J., & Dinero, T. E. (1981). A cross-cultural analysis of the fairness of the Cattell Culture Fair Intelligence Test using the Rasch model. *Applied Psychological Measurement*, *5*(3), 355-368. [https://doi.org/10.1177/014662168100500309](https://doi.org/10.1177%2F014662168100500309)

Radloff, L. S. (1977). The CES-D scale: A self-report depression scale for research in the general population. *Applied psychological measurement*, *1*(3), 385-401. [https://doi.org/10.1177/014662167700100306](https://doi.org/10.1177%2F014662167700100306)

Radloff, L. S. (1991). The use of the Center for Epidemiologic Studies Depression Scale in adolescents and young adults. *Journal of youth and adolescence*, *20*(2), 149-166.
